# Bilateral simultaneous tibial tubercle avulsion fractures in an adolescent baseball player: Case report and literature review

**DOI:** 10.1016/j.ijscr.2023.108986

**Published:** 2023-10-25

**Authors:** Marcantonio V. Pinci, Lucas De Virgilio-Salgado, Alexandra Claudio-Marcano, Joseph Salem-Hernandez, Daniel L. Castañón-Pereira, Humberto Guzman

**Affiliations:** a Orthopaedic Surgery Department, University of Puerto Rico, Medical Sciences Campus, San Juan, Puerto Rico; b University of Puerto Rico School of Medicine, University of Puerto Rico Medical Science Campus, San Juan, Puerto Rico; c San Juan Bautista School of Medicine, Caguas, Puerto Rico

**Keywords:** Tibial fracture, Bilateral avulsion fractures, Anterior knee pain, Athletic participation, Overuse injury, “Case report”

## Abstract

**Introduction and importance:**

Fractures in the proximal tibial tuberosity are rare injuries. Even more uncommon are bilateral simultaneous fractures. Due to the few cases reported in the literature, we aimed to present a case which may contribute to the diagnosis and treatment of bilateral simultaneous tibial tubercle fractures.

**Case presentation:**

A 13-year-old Hispanic male presented to the emergency department after experiencing sudden knee buckling while running after standing up from the catcher's position (squatted) during a baseball game, causing him to collapse to the ground. Plain radiographs revealed displaced tibial tubercle avulsion fractures in both knees. He underwent bilateral open reduction and internal fixation. Fracture healing was completed without complications.

**Discussion:**

To the best of our knowledge, this is the first documented case of a Hispanic pediatric baseball player, adding to the small number of reported cases of bilateral tibial tubercle fractures. The presented case is rare in terms of the mechanism of injury, which has been scantly reported in the literature.

**Conclusion:**

Due to the rarity of atraumatic bilateral tibial tubercle fractures we believe this documentation may be of clinical relevance.

## Highlights

• Atraumatic bilateral tibial tubercle avulsion fracture in pediatric baseball player
• Tibial tubercle avulsion fracture without history of Osgood-Schlatter disease
• Bilateral open reduction internal fixation in tibial tubercle avulsion fracture

## 1 Introduction

Tibial tubercle avulsion fractures are uncommon injuries in adolescents, accounting for 0.4 % to 2.7 % of pediatric fractures and less than 1 % of all physeal fractures [[1], [2], [3]]. Bilateral simultaneous avulsion lesions are extremely rare, with very few cases, less than 28 reported in the literature to date [1,3]. These injuries typically occur in male adolescents with an average age of 14 years old, before the proximal tibial physis closes [1]. The most common mechanism of injury involves jumping, with only 7 cases reporting running as the injury mechanism [3]. Predisposing factors include tight hamstrings, low patella, preexisting Osgood-Schlatter disease and Osteogenesis Imperfecta [4].

The proximal tibia has two ossification centers: a primary ossification center at the proximal tibial physis and a secondary ossification center at the tibial tubercle apophysis, which is the insertion site of the patellar tendon [5]. Physeal closure progresses from posterior to anterior and proximal to distal, which allows the distal secondary ossification center to be at higher risk of injury in older children [6]. These injuries are more prevalent among males due to increased quadricep strength, higher athletic participation, and later age at bony fusion [7,8].

Tibial tubercle avulsion fractures occur due to an eccentric force exerted by the knee extensor mechanism on a weakened unfused anterior proximal tibia physis [9]. Tubercle avulsion is thought to occur during aggressive knee flexion against a contracted quadriceps (i.e., landing from a jump) or powerful quadriceps contraction against a flexed foot (i.e., jumping) [6]. Both of these mechanisms are associated with sports that require jumping and sprinting, most commonly basketball and football [9]. These types of fractures are commonly categorized based on Ogden's classification system [16].

The Ogden classification is the most commonly used classification system and is a modification of the original Watson-Jones classification introduced in 1955. It is based on fracture location and displacement relative to the tibial physis. Type I are fractures of the secondary ossification center (near insertion of patellar tendon); type II are fractures between the first and second ossification centers; type III are coronal fractures with proximal extension through the joint. There have been many modifications to the Ogden classification system since its original development. Ryu and Debenham added a type-IV injury, where the fracture extends posteriorly through the entire proximal tibial physis [16]. McKoy and Stanitski then added a type-V injury, a periosteal sleeve avulsion of the patellar tendon insertion from the secondary ossification center [7]. The classification was subdivided into A (non-comminuted fractures) and B (comminuted fractures) [7].

Despite their rarity, clinicians should recognize that these fractures can result in significant damage to soft tissue, vascular compromise, intra-articular damage, genu recurvatum (caused by a growth arrest from the anterior tibia), bursitis (screw prominence), and even lead to compartment syndrome (caused by bleeding from the anterior tibial recurrent artery) [6,9].

We report the case of a 13-year-old Hispanic male without a previous history of Osgood-Schlatter disease (OSD), who presented to the emergency room of an academic center, after sustaining simultaneous atraumatic bilateral tibial tubercle avulsion fractures while running after standing up from a squatting position during a baseball game. This case has been reported in line with the SCARE 2020 criteria [10].

## 2 Case presentation

A 13-year-old Hispanic male with no past medical history presented to the emergency department with an inability to walk and severe pain in both knees. The patient experienced sudden knee buckling while running after standing up abruptly from the catcher's position (squatted) during a baseball game, causing him to collapse to the ground. There was no direct trauma to the knees during the event. The patient's height is 1.62 m and weight is 59 kg with a BMI of 22.5 kg/m^2^.

**Fig. 1:**
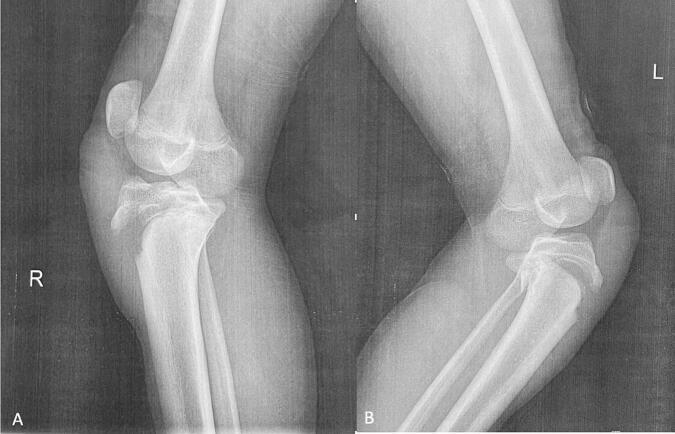
Pre-operative lateral plain radiographs of both knees. (A) Right tibial tubercule fracture, Ogden type lll. (B) Left tibial tubercule fracture, Ogden type lV.

Physical examination revealed an inability to bear weight, swelling over the bilateral tibial tuberosities, inability to fully extend either knee, and joint effusion. Gross examination found no neurovascular deficits. The patient had no prior history of musculoskeletal disease, such as Osgood-Schlatter disease, or any other knee-related issues. Laboratory testing for underlying metabolic or endocrine disorders was unremarkable. No predisposing factors were found. Anteroposterior and lateral plain radiographs of both knees revealed displaced bilateral tibial tubercle avulsion. CT imaging was performed on both knees to evaluate for intra-articular extension of the fractures. CT scan of the right knee demonstrated intra-articular extension with fracture lines extending to the proximal articular surface through the epiphyseal plate. CT imaging of the left knee revealed no intra-articular extension. The fractures were classified as Ogden type III in the right knee (Fig. 1A) and Ogden type IV in the left knee (Fig. 1B).

**Fig. 2:**
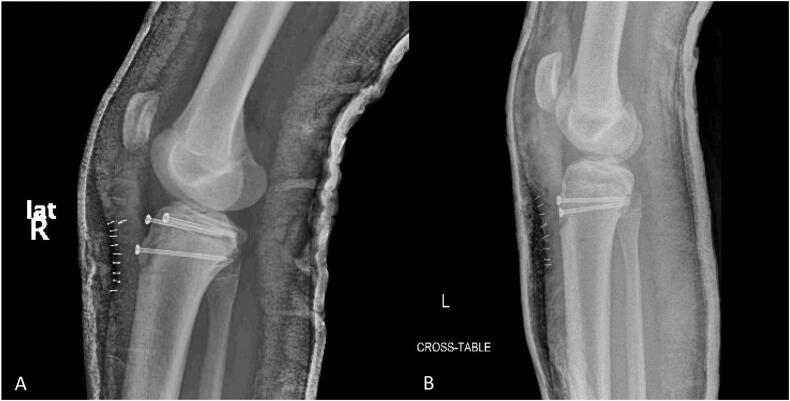
Immediate postoperative plain radiographs of both knees. (A) Right knee showing three 4.0 mm partially threaded cannulated screws. (B) Left knee showing two 4.0 mm partially threaded cannulated screws.

Due to the fact that operative treatment is indicated for type II-IV fractures [17], and our patient had a type III fracture on the right knee and type IV fracture on the left knee, surgical intervention was necessary for both fractures, which involved open reduction and internal fixation (ORIF) using screws. Since the patient did not present signs of compartment syndrome, he underwent surgery 24 h after the injury. After receiving intravenous (IV) antibiotics and spinal anesthesia, the patient was placed in a supine position and an anterior approach was made over the tibial tubercle on both knees. After soft tissue dissection and hematoma drainage, the fracture bed was cleared of interposed periosteum and the tibial tubercle fractures were reduced anatomically under direct visualization. The right knee (Ogden type III) was internally fixed with three partially threaded cannulated screws of 4.0 mm (Implants for Trauma Surgery [ITS], Austria). The left knee (Ogden type IV) was internally fixed with two partially threaded cannulated screws of 4.0 mm. The periosteum was sutured to the patellar tendon to reinforce the fixation (Fig. 2A–B). Both legs were initially placed in long cylinder splints and later exchanged for bilateral hinged knee braces.

Postoperatively, the patient's legs were immobilized with long cylinder splints and kept non-weight bearing for the first three weeks after surgery. He was then transitioned to hinged knee braces and physical therapy was then started with passive range of motion (ROM) and partial weight-bearing allowed. He continued working with a physical therapist and at six weeks after surgery, full weight-bearing and active ROM exercises with quadriceps strengthening were permitted. Physical therapy with focus on strengthening and increasing ROM continued until the patient regained strength, was back to full ROM and managed to walk without assistance.

At 14 weeks after surgery, the patient displayed great ROM (0°-120°) and regained bilateral knee extension strength. Full sporting activity was withheld until full ROM was achieved by the patient.

**Fig. 3:**
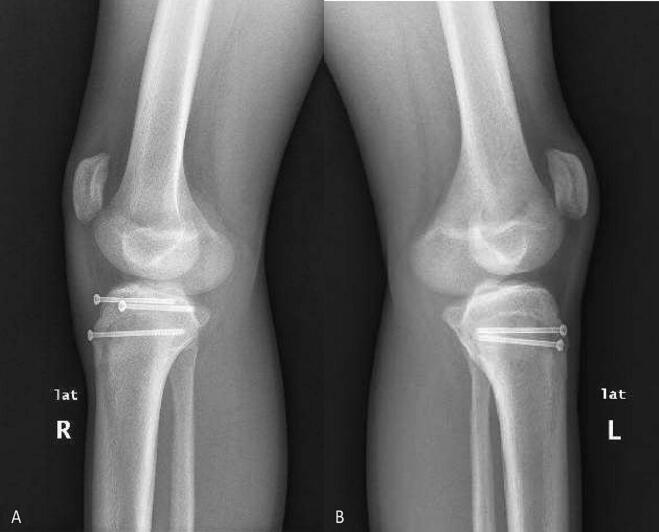
Six-month postoperative lateral plain radiographs of both knees. (A) Right knee showing three 4.0 mm partially threaded cannulated screws. (B) Left knee showing two 4.0 mm partially threaded cannulated screws.

Plain radiographs at six months after surgery demonstrated anatomic fracture reduction and alignment with screws in adequate position and fracture healing (Fig. 3A–B). The patient healed without complications and has regained full range of motion (0°–140°) in both knees, without limitations during routine or sporting activities. He has returned to playing baseball and is performing comparable to pre-injury levels.

## 3 Discussion

Bilateral simultaneous avulsion fractures of the tibial tuberosity are rare injuries [11]. Giunchi et al. recently reported two new cases emphasizing the importance of increasing awareness of this rare injury pattern to ensure prompt diagnosis and treatment [1]. This case about a pediatric Hispanic baseball player contributes to the small number of reported cases over the past 70 years [1].

Patients suffering from simultaneous bilateral tibial tuberosity avulsion fractures have a mean age of 14.6 years (range, 13 to 16y) and are predominantly male [12]. During this critical period in a young patient's development, the physis is undergoing physiologic changes that weaken its ability to resist tension loading, making it prone to injury [3]. Predisposing factors include tight hamstrings, low patella, preexisting Osgood-Schlatter disease and Osteogenesis Imperfecta, however our patient did not present any of these risk factors [13]. The available body of evidence points towards sports with high degrees of jumping and landing as the most common mechanisms driving this injury, with basketball and football being the most common [3,12].

This case, however, reports the rare occurrence of an atraumatic simultaneous bilateral tibial tuberosity avulsion in a baseball catcher, as the athlete began running after abruptly standing from a squatting position. When assessing our case, it is crucial to consider their role as a baseball catcher, which involves excessive periods of squatting. Baseball catchers are required to repetitively load their knees in a hyperflexed position for prolonged periods of time [14]. This consistent pattern of squatting puts a considerable amount of tension on the patellofemoral joint, leading to an increase in joint stress [15]. When the amount of force applied by the patellar tendon exceeds the strength of the physis, it succumbs to avulsion injury [3]. The accumulated stress load on their knees due to persistent and repetitive periods in the hyperflexed position might have been a contributing factor driving the mechanism of injury.

The primary goals of treatment include restoration of the extensor mechanism, anatomic reduction, and restoration of the joint surface, if it is involved [2]. Non-operative treatment may be appropriate for type-IA, IB, and IIA injuries, given that there is no significant displacement of the avulsed fragment and the extensor mechanism remains intact [17]. The closed reduction technique involves knee hyperextension and inline traction, which enables fragment alignment and congruity of the joint surface [18]. Following immobilization in a long leg or cylinder cast, patients are advised to avoid bearing weight for a duration of four to six weeks, after which they undergo rehabilitation [2].

In cases where there is significant fracture displacement or intra-articular extension, open reduction is needed to achieve anatomic reduction and restoration of the tibial joint surface [19]. In the absence of surgical fixation, the continuous pull of the patellar tendon can cause persistent fracture displacement, leading to fracture nonunion, malunion or improper healing [20]. ORIF using cannulated compression screws or closed reduction and percutaneous pinning (CRPP) are the treatment options for most fractures [2]. Periosteal suture repair may also be a supplemental fixation option for younger patients who have not developed adequate quadriceps power [2].

Based on the Ogden classification system our patient presented with a type III injury on the right knee and type IV injury on the left knee [16]. Due to the Ogden classification and displaced nature of both fractures, surgical management was indicated for both injuries. In unison with the cases reported by Elbaum and Giunchi, our patient was treated with ORIF using partially threaded canulated screws. While established guidelines and techniques are already available to address displaced fractures [2], it is noteworthy to highlight that the specific approach to treatment and surgical intervention may vary depending on fracture characteristics, as well as surgeon preference and expertise.

Although the bilateral nature of our patient's injury required him to be non-weight-bearing during the first three weeks after surgery, with the help of a physical therapist, he successfully progressed to full weight-bearing after six weeks. Six months after surgery the patient had regained full ROM, and his radiographs demonstrated anatomic fracture reduction and alignment with hardware in adequate position. Both fractures healed without complications, and he was able to return to his daily activities without any limitations. Furthermore, he reincorporated to his baseball team and is performing comparable to pre-injury levels.

## 4 Conclusion

We reported the first documented case of atraumatic bilateral simultaneous tibial tubercle avulsion fractures in a pediatric Hispanic baseball player. Although this is a rare type of fracture, our case serves as a reminder of the importance of having a high degree of clinical suspicion, which facilitates prompt diagnosis and surgical management of these injuries. Early identification and classification of these fractures allows for determination of the appropriate treatment alternative and a faster return to pre-injury levels. Consequently, this leads to better outcomes and increased patient satisfaction. Due to the rarity of this pathology, and importance of early diagnosis and management, we believe the dissemination of this case can be of clinical relevance and can contribute to the limited existing literature on the topic.

## Consent

The authors confirm that informed consent has been obtained from the involved patient or if appropriate from the parent, guardian, power of attorney of the involved patient; and, they have given approval for this information to be published in this article.

## Ethical approval

This study is a case report and does not require ethical from our institution's IRB. The patient was not physically involved in the research and only previously collected data was used. However, we have complied with HIPPA to protect the patient's information.

## Funding

This research did not receive any specific grant from funding agencies in the public, commercial, or not-for-profit sectors.

## Author contribution

Marcantonio V. Pinci, MD: Conceptualization, investigation, data curation, writing and editing.

Lucas De Virgilio-Salgado, MD: Investigation, data curation, writing, and editing.

Alexandra Claudio-Marcano, MD: Writing, data curation, reviewing and editing.

Joseph Salem-Hernandez, BS: Data curation and writing.

Daniel L. Castañón-Pereira, BS: Data curation and writing.

Humberto Guzman, MD: Investigation, supervision, reviewing, and editing.

## Guarantor

Marcantonio V. Pinci, MD and Alexandra Claudio-Marcano, MD; Department of Orthopaedic Surgery, University of Puerto Rico, Medical Sciences Campus, San Juan, Puerto Rico.

## Research registration number

Not applicable. This case report does not consist of a “First in Man case report.”

## Conflict of interest statement

The authors have no conflict to disclose.
